# Gender differences in safety issues during Fingolimod therapy: Evidence from a real‐life Relapsing Multiple Sclerosis cohort

**DOI:** 10.1002/brb3.804

**Published:** 2017-08-29

**Authors:** Alessia Manni, Vita Direnzo, Antonio Iaffaldano, Valentina Di Lecce, Carla Tortorella, Stefano Zoccolella, Pietro Iaffaldano, Maria Trojano, Damiano Paolicelli

**Affiliations:** ^1^ Department of Basic Medical Sciences, Neuroscience and Sense Organs University of Bari” Aldo Moro” Bari Italy

**Keywords:** fingolimod, gender differences, infections, liver function tests, multiple sclerosis

## Abstract

**Objective:**

Benefits and risks of new therapies in Multiple Sclerosis (MS) must be balanced carefully and tailored to patients. We aimed to describe our experience with Fingolimod (FTY), correlating demographics, clinical and hematological features of the Relapsing MS (RMS) cohort with the occurring Adverse Events (AEs).

**Material and Methods:**

Pretreatment screening tests, cardiological observation, and safety follow‐up data were analyzed in 225 RMS patients. Changes in continuous data were analyzed post hoc with Wilcoxon ranks test; categorical variables were examined using McNemar test. Two‐way repeated‐measures analysis of variance (ANOVA) was used to analyze differences between baseline characteristic of the cohorts and Liver Function Tests (LFT) alterations. Binary logistic regression models were used to identify which of the baseline factors influenced LFT alterations and the occurrence of infections.

**Results:**

During 2 years of follow‐up 24 patients (10%) interrupted FTY. Discontinuation most often was due to AEs (n = 14) or breakthrough disease (n = 5). The most frequently AEs were infections (10.6%). After the first year patients showing an infectious episode were mostly female (*p *= .04). The infections did not correlate with the decrease in white blood cells or to lymphocyte count. AST and ALT alterations ​​were observed mostly in males (*p *= .002 and *p *= .01, respectively), and increase in GGT ​​was reported in subjects older at FTY beginning (*p *< .05).

**Conclusions:**

For a patient‐centered safety monitoring of FTY, we may apply gender‐specific warnings, for the detection of transaminases abnormalities and infectious episodes.

## 1 INTRODUCTION

Treatments of Multiple Sclerosis (MS) underwent a double revolution over the past decades. The first revolution happened in 1993, with the interferon (IFN) β‐1b, the first therapeutic option for MS to modify the natural course of the disease. The second is represented by the oral route of administration of newer therapeutic options that promotes patient satisfaction and increases compliance.

Fingolimod (FTY) is the first oral disease‐modifying therapy (DMT) approved at 0.5 mg once daily for the treatment of relapsing forms of MS (US Food and Drug Administration (FDA), 2010; European Medicines Agency (EMA), 2011). FTY is a sphingosine 1‐phosphate (S1P) analog that, targeting the S1P pathway by regulation of lymphocyte trafficking from secondary lymphoid organs into the systemic circulation, inhibits lymphocyte egress from lymph nodes (Massberg & Von Andrian, 2006; Schwab & Cyster, 2007), whereas binding S1P receptors expressed in the CNS seems to modulate neurogenesis, neural function, and migration (Dev et al., 2008).

Two phase III clinical trials proved the efficacy of FTY therapy in MS (Cohen et al., 2010; Kappos et al., 2010).

S1P receptors are expressed differentially in various tissues, including the cardiovascular system (Blaho & Hla, 2011; Brinkmann, 2007): modulation of S1PRs on cardiac cells may explain the transient effects on heart rate (HR) and atrioventricular conduction, sometimes accompanied by a decrease in blood pressure (BP) (Budde et al., 2003; Cohen et al., 2010; Kappos et al., 2010; Laroni et al., 2014; Paolicelli et al., 2015).

Others organ‐specific adverse events (AEs), including elevation of liver function tests (LFT), headache, herpes infections, and macular edema, were observed in patients receiving FTY during clinical trials (Cohen et al., 2010; Kappos et al., 2010).

To gain a better understanding of suitable candidate for FTY therapy, further experiences of clinical practice can be useful for the overall management of patients. We hereby present our experience with FTY trying to delineate an individual characterization of the patient, to ensure proper treatment monitoring for each FTY‐treated patient.

## 2 METHODS

### 2.1 Patients, start‐up, and safety monitoring procedures

We observed 225 RR‐ MS FTY‐treated patients, followed at the Center for diagnosis and treatment of Demyelinating Diseases of the University of Bari, Italy. Clinical, therapeutic, and demographic characteristics of all patients were collected using the electronic database of iMed, a comprehensive, easily accessible database of information on MS patient's medical and treatment histories. The first administration of the drug took place between October 2013 and February 2015. The follow‐up period was 2 years ± 7 months: 174 patients were followed up to 6 months, 124 for at least 12 months, 112 up to the eighteenth month, and 97 were followed up for at least 24 months.

All subjects had a recent (within 6 months before treatment initiation) screening 12‐lead ECG with cardiologist interpretation and echocardiogram, complete blood count, and LFT. All patients were tested for anti‐Varicella Zoster Virus (VZV) IgG and IgM antibody titers. Patients also had a baseline ophthalmologic evaluation specifically looking for macular edema. Moreover, we asked all patients a complete dermatological evaluation with mole mapping to minimize the risk of skin cancer.

After the assessment of these warning area patients received the first dose and were observed with continuous ECG, and hourly measurement of HR and BP, for at least 6 hours, as required.

Complete blood counts were evaluated after 1, 3, and then every 3 months from the first administration of the drug and in case of reported symptoms or signs of infection. A lymphocyte count lower than 0.2 × 10 ^ 9 / l, if confirmed, led to the discontinuation of treatment. In the absence of clinical symptoms, LFT were monitored after 1, 3, 6, 9, and 12 months of treatment, and periodically thereafter: the treatment was discontinued if LFT reached values 5 times higher than the normal range, stable in subsequent follow‐ups. AEs were reported at their occurrence or in the scheduled visits. Moreover, after three months from treatment beginning, patients were assessed for the self‐report Patient Global Impression of Change (PGIC), to reflect their belief about the efficacy of treatment.

### 2.2 Compliance with ethical standards

The protocol of this study was approved by the local Institutional Review Board of the University of Bari, Italy, and all subjects signed informed consent to treatment.

### 2.3 Statistical analysis

Clinical and demographical features of patients were reported as mean ± standard deviation (SD) for continuous data (lymphocytes, white blood cell, HR values, and PGIC values), or as relative and absolute percentage for categorical variables.

Changes in continuous data were analyzed post hoc with the Wilcoxon ranks test; similarly categorical variables were examined using the McNemar test. Two‐way repeated‐measures analysis of variance (ANOVA) was used to analyze differences between baseline characteristic of the cohorts and LFT alterations at 3, 6, and 12 months of therapy (Sheldon et al., 1996; Sheskin, 2004). Binary logistic regression models were used to identify which of the baseline factors influenced LFT alterations and the occurrence of infections.

The most frequent AEs were detected in terms of relative and absolute incidence.

Significance for all tests was defined as *p *< .05.

All analyses were performed with SPSS software version 17.0.

## 3 RESULTS

Baseline characteristics of the cohort are shown in Table 1.

**Table 1:** Baseline characteristics of the RMS fingolimod‐treated cohort

Total patients	*n* = 225
Gender	
M	71 (31.6%)
F	154 (68.4%)
Age at Fingolimod beginning	
Mean(±SD)	39.6 (± 9.5)
Time from MS onset to Fingolimod start—years	
Mean (±SD)	12.8 (±7.7)
No history of DMDs—%	1.8 (4)
EDSS pre‐Fingolimod	
Median (range)	3.5 (1.0–6.0)
Relapse rate within the previous year	
Mean (±SD)	0.7 (± 0.6)

### 3.1 First dose observation (FDO) and cardiac safety

Of the 225 patients, none had a history of heart disease that contraindicated FTY. Thirty‐one (13.8%), however, had comorbidity that could influence their cardiological safety: nine hypertension treated with ACE inhibitors, angiotensin receptor blockers, beta blockers, and diuretics in two, three, one, and three patients, respectively); two diabetes (one treated with long‐acting insulin and the other with sulfonylureas); and 20 Thyroid disorders (14 of whom were treated with levothyroxine).

FDO was uneventful in 152 patients (67.6%). The other 73 patients showed transient ECG abnormalities during the 6‐hour monitoring, mostly asymptomatic. We needed to prolong the cardiac monitoring for six patients (2.7%). Three of them had a prolonged QTC, and prolonged their ECG monitoring for 3 hours; in the other three cases it was necessary to extend the observation period to 8 hours for the presence of a difference pre/posttreatment of 22 bpm in one patient, for a persistent bradycardia in the second one, and for the occurrence of an incomplete right bundle branch block with symptoms of malaise in the third one. Moreover, for a single patient it has been necessary to discontinue the treatment for the occurrence of a symptomatic II degree Atrio‐Ventricular block (AVB).

Significant decreases in HR were observed 6 hours after FDO (−9.7 ± 9.3 bpm).

Considering the effect of FTY on QT interval, at the end of monitoring we found a reduction of 4.4 ± 15.8 msec (predose QTc: 424.2 ± 18.8 msec, postdose QTc: 418.5 ± 19.9 msec).

Monitoring of BP has led to the observation of an average reduction, at the end of the FDO, of 4.9 ± 12.3 mmHg in systolic and 3.8 ± 10.4 mm Hg in diastolic BP.

Vital signs remained within normal standards after long‐term follow‐up, apart from 10 cases that showed a persistent increase in BP, which in five cases needed to be treated. From the 3rd month of follow‐up a 54‐year‐old woman showed an atrial fibrillation that needed to be treated with beta blockers, but this not lead to the drug discontinuation.

### 3.2 Overall safety

Twenty‐four patients (10.7%) definitively interrupted FTY treatment (Table 3). One of these was a woman who experienced a pregnancy after 2 years of FTY exposure; the pregnancy resulted in the birth of a healthy newborn.

During the observation period, 72 patients (32%) reported at least one AEs.

**Figure 1:**
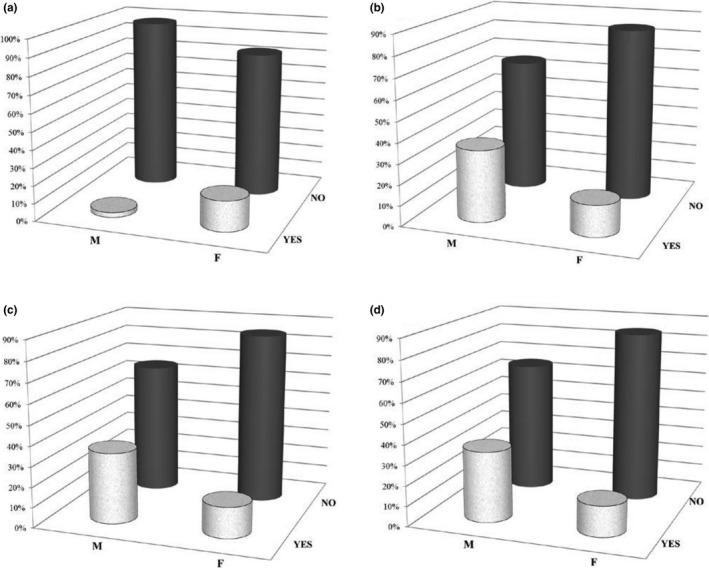
Occurrence of Infections after the first year of treatment (a); occurrence of AST alterations (b); ALT alterations (c); and GGT alterations (d) at the 6th month of therapy

The most frequently reported clinical AEs are summarized in Table 2. The most common AEs were infections: 24 (10.7%) patients had an infection during FTY treatment, mostly on the upper respiratory and urogenital tracts, generally mild. Patients showing an infectious episode after the first year of therapy (8.8%) were mostly female ones (*p *= .04), as shown in Figure 1a. These data were confirmed at the binary logistic regression (*p *= .041, OR=9.45, CI = 1.9–2.8). Three of these patients experienced a VZV infection, which led to the interruption of the drug. No serious sequelae exitated from these events. Infective episodes in these patients did not correlate with the total White Blood Cells (WBC) count or with the number of total lymphocytes.

**Table 2:** Most frequent clinical Adverse Events (AEs)

Clinical AEs	Incidence
Ophthalmological	Total 1
Macular imbibition	1
Cardiovascular	Total 11
Hypertension (treated)	10 (5)
Atrial fibrillation	1
Infections	Total 24
Urogenital tract	13
Upper respiratory tract	4
Herpes Zoster	3
Recurrent herpes labialis	2
Other skin infections	2
Dermatological	Total 12
Rashes	7
Acne worsening	2
Nail dystrophy	1
Hair thinning	1
Alopecia	1
Headache	Total 5
Weight loss	Total 5
Epigastralgia	Total 4
Epistaxis	Total 2

Ophthalmology evaluations were mostly uneventful: we observed one case of asymptomatic small increase in macular thickness at the third month; we strictly monitored retinal and macular volume, and we observed a spontaneous recovery at the sixth month follow‐up, with no need of treatment interruption.

Dermatological evaluation did not show serious AEs: the most reported were rushes, from mild to moderate intensity.

### 3.3 Effects on LFT

Patients underwent periodic (every 3 months) measurement of LFT in the same laboratory. We recorded an increase in LFT, lower than 5 times the normal range, in 13.3% of the cohort at the 3rd month of follow‐up, in 17.24% of patients at the 6th month follow‐up, and in 21.4% of patients at the 12th month follow‐up.

We tried to better characterize this subgroup of patients who developed LFT alterations, and we found that at the 3rd month follow‐up an increase in Aspartate aminotransferase (AST) values was observed in 11 patients (4.9%), who were older at FTY beginning (44.4 ± 10.3 vs. 37.7 ± 9.4, *p *= .009). We observed an increase in Alanine aminotransferase (ALT) values in 21 patients, mostly males (22.7% vs. 4.4%, *p *< .0001); also Gamma‐glutamyl transpeptidase (GGT) increased in males (21.2% vs. 11.7%, *p *= .06) older at the FTY beginning (44.6 ± 8.5 vs. 37 ± 9.3, *p *< .0001) and at disease onset (31.3 ± 9.7 vs. 24.4 ± 7.5, *p *< .0001).

After 6 months (Figure 1b–d) alterations of the AST values occurred predominantly in males patients (17.4% vs. 2.5%, *p *= .004); ALT alterations were observed in patients males (42.1% vs. 6.1% *p *< .0001) and older at disease onset (30.3 ± 9.6 vs. 25.04 ± 8, *p *= .005); GGT alterations were detected in patients older both at FTY beginning (43.2 ± 9 vs. 36.6 ± 9 *p *< .0001) and at disease onset (31.1 ± 9.7 vs. 24.6 ± 9.7 p<0.0001) and also males (35% vs. 15.2% *p *= .03).

At the 12th month, AST and ALT alterations were observed mostly in males (28.6% vs. 3.5%, *p *= .002 and 38.41% vs. 10%, *p *= .01, respectively). Alterations of GGT values were observed in subjects older both at FTY beginning (40.4 ± 8.3 vs. 36.6 ± 9.3, *p *< .05) and at disease onset (28.3 ± 8.67 vs. 24.7 ± 7.8, *p *= .04). These last data remained stable at the 24th month of follow‐up.

The binary logistic regression models confirmed the sex as the predictive variable responsible for raise in LFT.

### 3.4 Changes in the white blood cells count and lymphocyte

The WBC count significantly dropped after the 1st month of therapy (from 6927.29 ± 2048.3 to 4604.35 ± 1423.9; *p *< .0001), with a reduction of 31% ±17.3. A not statistically significant reduction was also observed from the 1st (4604.35 ± 1423.9) to the 6th month (4225.9 ± 1297.48), while from the 6th to the 12th month the WBC counts tend to rise even without reaching statistical significance (4510.44 ± 1492.44).

Considering the absolute number of lymphocytes at FTY beginning, we observed a statistically significant reduction both after 1 (*p *< .0001, with a decrease of 65.9%) and 6 months of therapy (*p *= .01, lymphocytes decreased of 67.9%). From the 6th to the 12th month we noticed a not statistically significant increase in the total number of lymphocytes.

We reported three cases of severe lymphopenia that lead to the drug discontinuation (Table 3).

**Table 3:** Reasons for Fingolimod discontinuation

	Number of patients: 24 (10.6%)	Month of discontinuation
Increase in Liver Function Test	5	5th, 7th, 8th, 9th, 13th
Lack of compliance	4	9th, 13th, 18th, 22nd
Persistence of MRI activity	3	12th, 13th, 18th
Lymphopenia	3	6th, 8th, 13th
VZV reactivation	3	9th, 13th, 18th
Persistence of clinical activity	2	12th, 16th
Symptomatic II degree Atrio‐Ventricular block	1	FDO
Progressive anemia	1	3rd
Increase in amylase	1	6th
Pregnancy	1	25th

### 3.5 Patient‐reported outcomes

Considering their own believes about the efficacy of the treatment, the major part of the study population (41.8%), after the 6th month of therapy rated, at the PGIC scale a mark of 6 (better, and a definite improvement that has made a real and worthwhile difference), as shown in Table 4. This perception did not show statistically significant variation at the 1st and 2nd year of follow‐up.

**Table 4:** PGIC evaluated at the 6th month follow‐up

	Incidence (%)
1 = no change or worsened condition	‐
2 = almost the same, hardly any change	11 (6.3)
3 = a little better, but no noticeable change	14 (8.1)
4 = somewhat better, but the change has not made any real difference	19 (10.9)
5 = moderately better, and a slight but noticeable change	41 (23.6)
6 = better, and a definite improvement that has made a real and worthwhile difference	72 (41.4)
7 = a great deal better, and a significant improvement that made the difference	17 (9.8)
Total	174 (100)

## 4 DISCUSSIONS AND CONCLUSIONS

Analyzing real‐life experiences of safety outcomes may be very difficult because of the small sample sizes usually provided that can overestimate the rate of rare AEs, but also because of the underreporting bias by physicians.

In this work we summarized our 2 years experience of FTY use, focusing on safety and tolerability issues in a cohort of 225 RMS patients.

We discussed in our previous work how the cardiac safety profile was confirmed good in a “less selected” population that for baseline characteristic and comorbidities could better represent the reality that clinicians have to face everyday (Paolicelli et al., 2015). We preferred to discontinue the drug after the occurrence of the second‐degree AVB, but other postmarketing experiences demonstrated that patients with second‐degree AVB could continue the drug, being monitored until resolution of these events, as these may persist several days after the first FTY administration (Saccà et al., 2016).

One of the “warning area” of FTY‐treated patients is macular edema, reported in 0.6% patients in clinical trials (Kappos et al., 2014), mostly in the first 6 months of therapy, and in most cases with recovery after drug interruption. In our experience, only a mild case of macular succulence was detected in a patient without comorbidities at the 3rd month of treatment, but it resolved spontaneously. According to the label, patients with diabetes mellitus or a history of uveitis are at increased risk of macular edema, so should have regular follow‐up examinations. Basing also on evidence from others case reports (Li et al., 2014), we agree that clinician may wish to consider continuation of FTY therapy under close monitoring in patients who have stable vision but macular changes, with special attention to those patients with comorbidities that increase this risk.

Although the incidence of malignancies in clinical trials was similar in the FTY 0.5 mg and in the placebo arms, reports of skin malignancies in Phase II MS trial resulted in active screening for skin cancers. FTY impact on mechanisms involved in melanoma formation and progression remains uncertain: in addition to the potential direct effects of FTY on melanocytes, immunomodulation—desirable in RR MS, but deleterious in tumor defense— may contribute to melanoma formation. However, the incidence of melanoma occurring in FTY‐treated patients is similar to the general population (almost 0.3%). In our study, no report of skin malignancies was found. Self‐examination of the skin every 3 months and a dermatologic full‐body skin evaluation annually may be the best management strategy during FTY therapy because early removal of melanoma lesions is the only curative treatment.

FTY overall incidence of infections was similar to placebo in clinical trials, with a slightly higher incidence of lower respiratory tract infections (Kappos et al., 2014). In our study, urogenital infections were the most frequently detected (13 of 24, 54%).

Although FTY leads to the retention of lymphocytes in the lymphnodes, the lymphocytes remain functional and retain their ability to proliferate and differentiate, as might happen in response to viral or bacterial attack; moreover, peripheral lymphocyte counts represent only approximately 2% of the total body stores of lymphocytes. However, some questions remain regarding herpes infections. Antibody status to Varicella zoster virus should be checked before starting FTY, but this could not always be enough: we experienced three cases of reactivation of VZV virus, even if all these patients had been tested for VZ antibodies, resulting in a high titer immunization. They all had previous infection during their childhood and did not report any history of recurrent VZV disease. The confirmation of VZV infection was followed by prompt initiation of antiviral treatment with acyclovir and discontinuation of FTY.

FTY has been shown to attenuate the antiviral immune response in MS patients as evidenced by reduced numbers of IFN‐γ‐producing T cells and decreased VZV‐specific proliferation of CD4+ and CD8+ T cells (Ricklin et al., 2013). In addition, frequencies of VZV‐specific memory T cells are reportedly low and amount to only half of HSV‐ and to less than a tenth of CMV‐specific T‐cell frequencies in immune adults (Asanuma et al., 2000; Tyler, 2015). Together, this may add to a disrupted defense against VZV reactivation from latency. Indeed, subclinical reactivation as determined by detection of VZV in saliva is a common consequence of fingolimod treatment (Ricklin et al., 2013). Prevention and management of VZV infections, predominantly herpes zoster, in adults with MS receiving newer DMTs, is a topic of clinical relevance: symptomatic reactivation of latent VZV has been reported in patients who received Monoclonal Antibodies (natalizumab, alemtuzumab) (Coles et al., 2012; Fine, Sorbello, Kortepeter, & Scarazzini, 2013), and prophylactic aciclovir can reduce the proportion of patients who had herpes zoster (HZ) (Coles et al., 2012). Current data do not support the general use of antiviral prophylaxis in patients receiving FTY given the low incidence of HZ infection (Arvin et al., 2015).

We noticed that having an infective episode did not correlate with the contingent total WBC count or with the number of total lymphocytes, confirming data from previous studies (Francis et al., 2014). The relative sparing of T‐effector memory cells, which are important in immune surveillance, and the preservation of function of sequestered lymphocytes, suggest that key features of the immune system are maintained during FTY therapy. However, considering the infections occurred within the first year of therapy, we noticed that patients with infection (5.2%) showed at the baseline, a lower number of white blood cells compared to “infections‐free patients” (5735.56 ± 1433.4 and 6982 ± 2057.83, respectively) and a lower number of lymphocytes (1717.8 ± 380.4 and 2116.6 ± 681.5, respectively), but these differences did not reach statistical significance .

Patients showing an infectious episode after the first year of therapy were mostly female ones. Usually, it has been proven that females tend to mount a stronger immune response that helps them to clear infections faster and reduces the risk of persistence; although this response helps to fight infecting pathogens, it makes women more susceptible to immune pathologies (Fish, 2008; Klein, 2012; Úbeda & Jansen, 2016). The immune system remodulation under FTY therapy may be partly responsible for this unexpected vulnerability of female patients to pathogens in our study. These findings show the need for future research with a larger sample.

Interestingly, we also recorded an increase in LFT, lower than 5 times the normal range, in 21.4% of patients at the 12th month follow‐up; but only five of our patients had to discontinue treatment because of elevation in LFT above 5 times the upper limit.

This finding in the pivotal trials occurred in a lower percentage of patients (0.5%) and only at the dose of 1.25 mg. We noticed that LFT monitoring may require a gender‐specific and age‐specific approach: male patients and those older both at FTY beginning and at the disease onset were most frequently interested by transaminases elevation, which in our cohort was detected from the third month of therapy. In vitro studies with human liver microsomes and animal studies have shown that cytochrome P450 (CYP) 4F2 and other enzymes of the CYP4F subfamily, with a minor contribution reported from other CYP enzymes, are largely responsible for the oxidative metabolism and the subsequent elimination of FTY (Jin et al., 2011; Kalsotra, Anakk, Boehme, & Strobel, 2002; Kovarik et al., 2005, 2006; Wang & Strobel, 1997; Waxman, Ram, Pampori, & Shapiro, 1995; Zimmerlin & Patten, 2000). Sex‐dependent expression of cytochromes P450 and their influence on drug metabolism and drug toxicity have been previously reported. Animal experiments, principally in rats, have revealed that expression of the number of P450 enzymes is sex dependent, with a considerably higher protein expression in female liver (Kalsotra et al., 2002; Wang & Strobel, 1997; Waxman et al., 1995) and this may justify our findings.

One of our patients experienced pregnancy during FTY exposure, resulted in the birth of a healthy newborn. However, the number of patients becoming pregnant during FTY treatment is too small to allow conclusions about its fetal safety. Considering the known risks of teratogenicity in animals and pregnancy outcomes reported from clinical studies, women should use effective contraception during FTY therapy and for 2 months after discontinuation.

Selecting a drug to treat MS is a complex process, in which comorbidities, family planning, and patients’ lifestyles must also be considered. Generally, FTY has a positive impact on patients’ lives and in our experience too, as our patients considered that the drug was a “better, and a definite improvement that has made a real and worthwhile difference” in their PGIC rating.

A careful evaluation of patients prior to initiating therapy, and a tailored monitoring thereafter, can reduce the risk of complications. Our study may highlight the need to apply a gender‐specific approach to FTY monitoring procedures: we suggest that particular attention must be provided to male patients, older at disease onset and at the FTY beginning, for the detection of LFT abnormalities; female patients, instead, must be checked for signs and symptoms of infections for a longer period, and of course, must be warned about the potential teratogenic effect of the drug. Further study will better clarify if these management strategies could enter in everyday clinical practice, addressing one of the most challenging areas in MS: personalized medicine.

## CONFLICT OF INTEREST

None declared.
